# Central Retinal Vein Occlusion With Signs of Ocular Hypoperfusion After Parent Artery Occlusion for a Traumatic Carotid-Cavernous Fistula: A Case Report

**DOI:** 10.7759/cureus.117891

**Published:** 2026-10-13

**Authors:** Yuki Kato, Ryusuke Hashimoto, Takayuki Baba

**Affiliations:** 1 Department of Ophthalmology and Visual Science, Chiba University Graduate School of Medicine, Chiba, JPN

**Keywords:** carotid-cavernous fistula, central retinal vein occlusion, macular edema, ocular hypoperfusion, parent artery occlusion, reduced retinal capillary perfusion, spontaneous retinal arterial pulsation, widefield fluorescein angiography

## Abstract

A carotid-cavernous fistula (CCF) can cause a wide range of ocular complications, including venous stasis retinopathy, secondary glaucoma, and central retinal vein occlusion (CRVO). Retinal complications have been reported after endovascular closure of the shunt, but there are few reports describing the ophthalmic course of an eye in which the shunt persisted and in which parent artery occlusion of the internal carotid artery was required.

A 63-year-old man was diagnosed with a traumatic right internal carotid artery CCF after a road traffic injury and underwent endovascular treatment on the second and third days after the injury. Because both the transvenous and the transarterial approaches were unsuccessful, parent artery occlusion of the right internal carotid artery was performed. According to the report from the referring institution, occlusion of the shunt was incomplete, and shunt flow into the right superior ophthalmic vein persisted. Angiography approximately six months after the injury confirmed the residual shunt, and no further endovascular treatment was considered feasible. Two months later, the patient noticed decreased vision in the right eye, presented to a local ophthalmologist, and was referred to our department.

At presentation, best-corrected visual acuity was 0.2 (logMAR 0.70) in the right eye, and the intraocular pressure (IOP) was 14 mmHg. The right eye showed dilated episcleral vessels, dilated retinal veins, attenuated retinal arteries, retinal hemorrhages extending from the posterior pole to the periphery, and marked macular edema, but venous tortuosity was not prominent. The pupillary light reflex was reduced, and pharmacologic mydriasis was poor, without posterior synechiae or iris atrophy. Spontaneous pulsation of the central retinal artery was present in the affected eye and was augmented by gentle digital pressure, whereas no pulsation was seen in the fellow eye. On widefield fluorescein angiography, dye first entered the central retinal artery 20.1 seconds after the start of injection and complete arterial filling was reached at 25 seconds; numerous microaneurysms were seen from the posterior pole to the mid-periphery, with widespread vascular leakage and reduced perfusion of the retinal capillary network. The fellow eye showed only a few microaneurysms and scattered hard exudates, consistent with mild nonproliferative diabetic retinopathy. The patient had type 2 diabetes mellitus, untreated for approximately 10 years and restarted after the current injury, and hypertension.

An intravitreal injection of an aflibercept biosimilar 2 mg was given on the day of presentation, followed by panretinal photocoagulation (PRP) delivered in four sessions for a total of 1,580 burns. Twenty-five days after the injection, the macular edema had clearly decreased but had not completely resolved, and it had recurred by day 67. Throughout the course, the IOP remained consistently lower in the affected eye than in the fellow eye. No iris or angle neovascularization was observed.

In this eye, findings of CRVO coexisted with findings suggesting reduced ocular perfusion pressure. Even in a patient with systemic risk factors, local factors should be considered when the findings are confined to, or markedly more severe in, one eye. When no further treatment of the causative lesion is possible, management of the ocular complications is limited to ophthalmic measures, and their effects and limitations must be evaluated separately.

## Introduction

A carotid-cavernous fistula (CCF) is an abnormal shunt between the internal or external carotid system and the cavernous sinus and is classified as traumatic or spontaneous. When reflux into the superior ophthalmic vein occurs, a wide range of ocular signs may develop, including dilated episcleral vessels, proptosis, raised intraocular pressure (IOP), and venous stasis retinopathy. In a multicenter retrospective study of patients with ocular manifestations, conjunctival hyperemia was present in 92.9%, proptosis in 50.0%, retinal hemorrhage in 50.0%, and retinal venous dilatation in 42.9%, and raised IOP (maximum 22-55 mmHg) was noted in 64.3% [1].

Central retinal vein occlusion (CRVO) is one of the serious ocular complications of CCF. Previous reports describe improvement of the retinal circulation and visual acuity after closure of the shunt [2], delayed development of CRVO after embolization [3,4], and macular edema that resolved with an anti-vascular endothelial growth factor (VEGF) agent after successful embolization [5,6]. In all of these, the shunt had been closed.

In contrast, there are few reports describing the ophthalmic findings and the extent to which they can be managed in an eye in which the shunt persisted and in which parent artery occlusion of the internal carotid artery was required. Depending on the extent of the occlusion, antegrade flow to the ophthalmic artery may be lost, and ocular perfusion may then depend on collaterals from the external carotid system, so that arterial hypoperfusion may become an issue in addition to the venous pathology.

We report a case in which the shunt persisted after endovascular treatment of a traumatic CCF, no further endovascular treatment was considered feasible, and findings suggesting reduced ocular perfusion pressure coexisted with findings of CRVO, accompanied by rapid visual loss.

## Case presentation

All dates are given relative to the day of the first visit to our department, which is designated day 0.

Background

A 63-year-old man sustained a road traffic injury 251 days before presentation and was found to have an intracranial hemorrhage. Head magnetic resonance imaging and magnetic resonance angiography established the diagnosis of a traumatic right internal carotid artery CCF. Cerebral angiography two days after the injury demonstrated a shunt at the cavernous segment of the right internal carotid artery with reflux into the right superior ophthalmic vein and the right superficial middle cerebral vein. Transvenous closure of the shunt was attempted, but the shunt point could not be reached, and transarterial embolization was also considered difficult because the feeding vessels were small. Three days after the injury, a right superficial temporal artery-middle cerebral artery (extracranial-intracranial) bypass was performed, followed by parent artery occlusion of the right internal carotid artery and transarterial embolization. According to the report from the referring institution, occlusion of the shunt was incomplete, and shunt flow into the right superior ophthalmic vein persisted.

Cerebral angiography approximately six months after the injury showed a residual pouch around the right cavernous sinus, with small feeding vessels from the external carotid system and persistent shunt flow into the right superior ophthalmic vein. At that time, no further endovascular treatment was considered feasible because of the vascular anatomy.

The angiographic findings described above are based on the report from the referring institution and were not independently reviewed by the authors.

Systemic history

The patient had type 2 diabetes mellitus and hypertension. He had been treated with insulin and metformin approximately 10 years earlier but had discontinued treatment on his own and had subsequently been untreated for a long period. Diabetes treatment was restarted at the referring institution after admission for the current injury, and at presentation to our department, he was receiving repaglinide 0.5 mg three times daily and dulaglutide 0.75 mg weekly. At presentation, the glycated hemoglobin was 7.2%, and the blood pressure was 150/90 mmHg. He had smoked 20 cigarettes per day from the age of 20-45 years. There was no history of allergy.

Course before referral to our department

After it had been decided that no further endovascular treatment was feasible, the patient was referred by the neurosurgical department to a local ophthalmologist and was examined 62 days before presentation to us. Best-corrected visual acuity (BCVA) in the right eye was 0.7 (logMAR 0.15), the IOP was 17 mmHg, and there was 4 mm of proptosis with dilated episcleral vessels. Fundus examination showed retinal hemorrhage and mild macular edema. Kinetic perimetry showed a left inferior quadrantanopia.

The patient subsequently noticed decreased vision in the right eye and was re-examined by the same ophthalmologist three days before presentation to us. The dilated episcleral vessels were unchanged, and increased retinal hemorrhage and marked macular edema were noted, prompting referral to our department. No ocular treatment was given during this interval.

Findings at presentation (day 0)

The ophthalmic findings at presentation are summarized in Table 1.

**Table 1:** Ophthalmic findings at presentation (day 0), before any treatment.

Finding	Right eye (affected)	Left eye
Best-corrected visual acuity (decimal/logMAR)	0.2/0.70	1.2/-0.08
Intraocular pressure (mmHg, non-contact tonometer)	14	15
Pupillary light reflex	Reduced	Normal
Response to mydriatic agent	Poor	Normal
Posterior synechiae/iris atrophy	Absent	Absent
Dilated episcleral vessels	Present	Absent
Lens opacity	Grade 0	Grade 0
Spontaneous pulsation of the central retinal artery	Present	Absent
Pulsation on digital pressure	Augmented	Not induced
Intraretinal microvascular abnormalities	Absent	Absent
Retinal venous dilatation	Present	Absent
Retinal venous tortuosity	Not prominent	Absent
Attenuation of retinal arteries	Present	Absent
Retinal hemorrhage	Posterior pole to periphery	Absent
Macular edema	Marked	Absent
Hard exudates	Absent	Nasal to inferior to the disc
Filling of the central retinal artery (from start of injection)	First appearance 20.1 s, complete filling 25 s	Not assessed
Microaneurysms	Numerous, posterior pole to mid-periphery	Approximately six at the posterior pole
Reduced retinal capillary perfusion	Widespread	Absent
Enlargement of the foveal avascular zone	Not appreciable	Absent
Retinal/iris neovascularization	Absent	Absent

BCVA was 0.2 (logMAR 0.70) in the right eye and 1.2 (logMAR -0.08) in the left eye. The IOP measured with a non-contact tonometer was 14 mmHg in the right eye and 15 mmHg in the left eye. Dilated episcleral vessels were present in the right eye (Figure 1).

**Figure 1:**
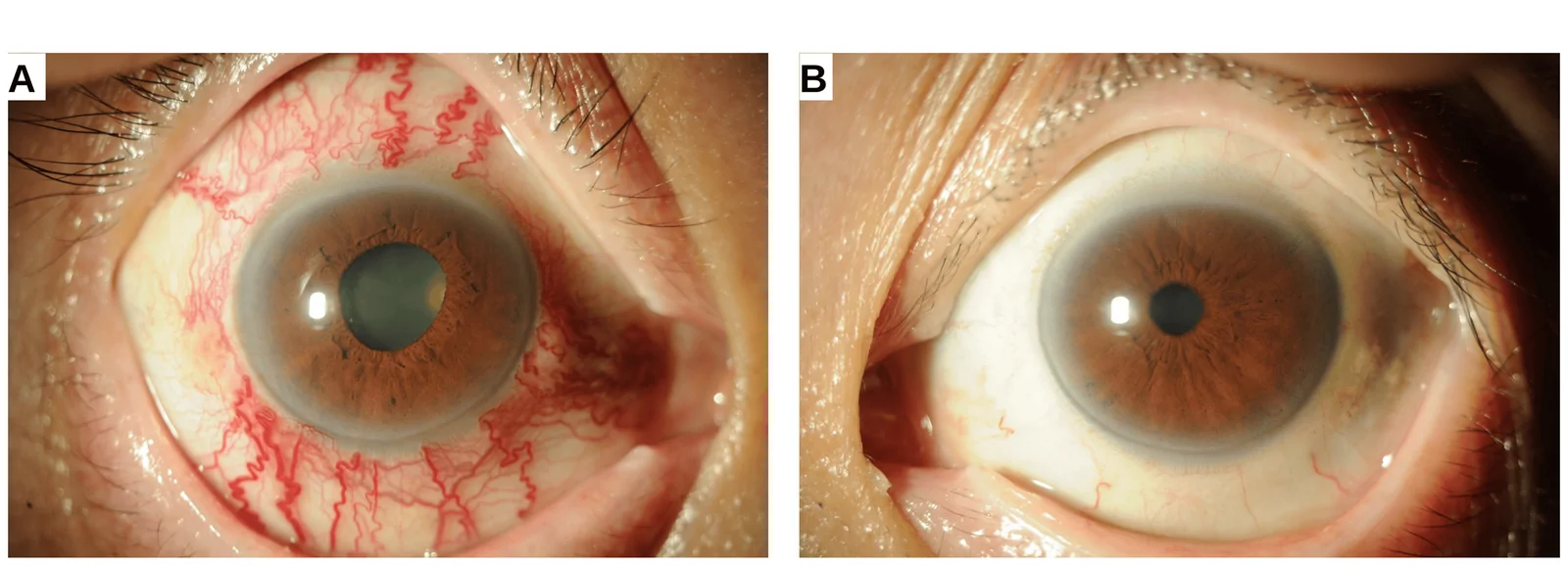
Anterior segment photographs at presentation. (A) The right eye shows dilated and tortuous (“corkscrew”) episcleral vessels, consistent with arterialized orbital venous drainage. (B) The left eye is unremarkable.

The pupillary light reflex was reduced in the right eye, and mydriasis with a fixed combination of tropicamide and phenylephrine was poor in that eye, without posterior synechiae or iris atrophy. The pupillary light reflex and mydriasis were normal in the left eye. Lens opacity was grade 0 in both eyes.

Fundus examination of the right eye showed dilated retinal veins without prominent tortuosity and attenuated retinal arteries. Retinal hemorrhages extended from the posterior pole to the periphery and were accompanied by marked macular edema. No hard exudates were seen. There was no retinal neovascularization, and undilated slit-lamp examination and gonioscopy showed no iris or angle neovascularization (Figure 2).

**Figure 2:**
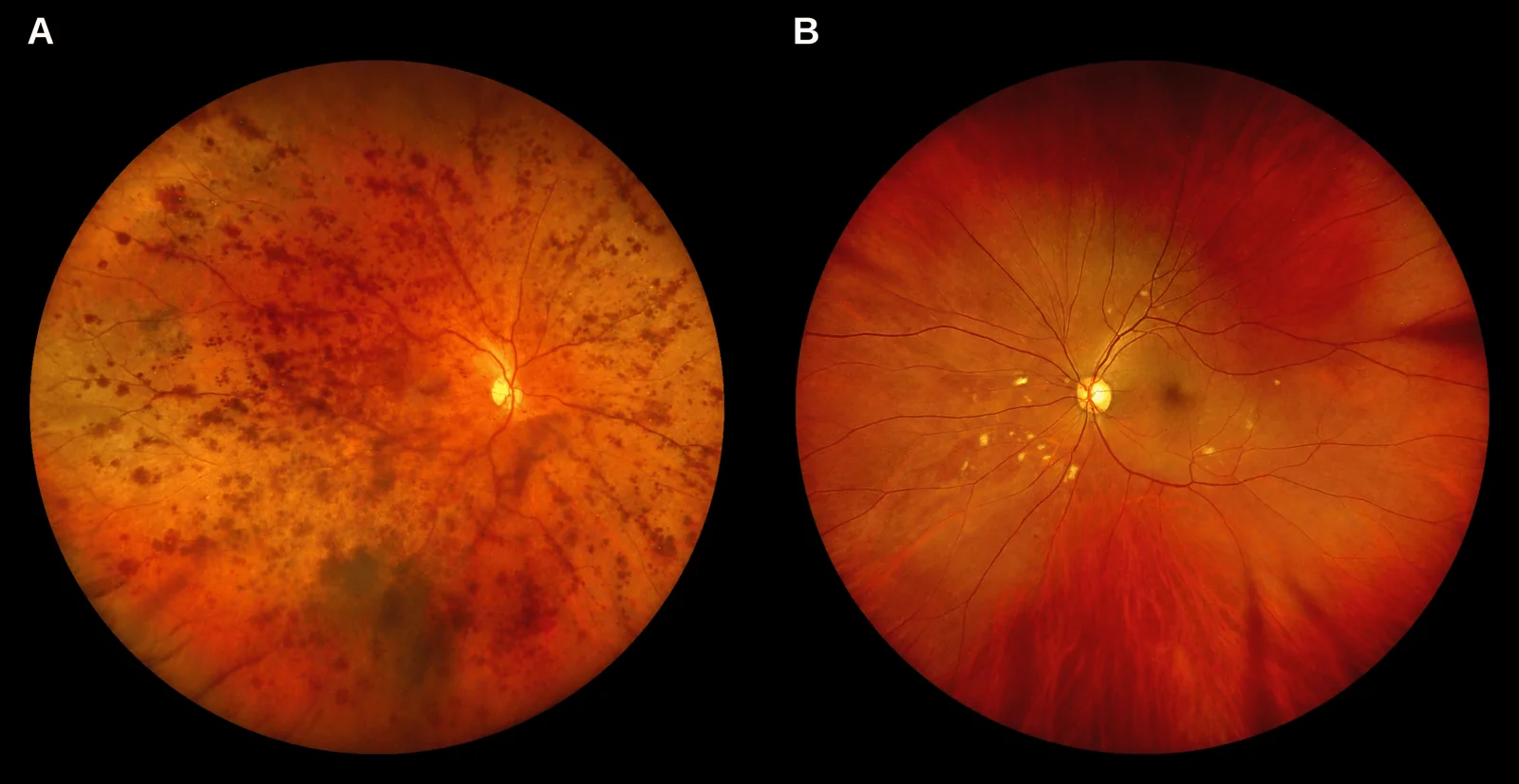
Color fundus photographs at presentation. (A) The right eye shows dilated retinal veins without prominent tortuosity, attenuated retinal arteries, retinal hemorrhages extending from the posterior pole to the periphery, and marked macular edema. (B) The left eye shows scattered hard exudates and a few microaneurysms, consistent with mild nonproliferative diabetic retinopathy, without hemorrhage, venous dilatation, or arterial attenuation.

Under pharmacologic mydriasis, slit-lamp examination showed spontaneous pulsation of the central retinal artery in the right eye, which was augmented by gentle digital pressure on the globe. No spontaneous pulsation was present in the left eye, and no pulsation was induced by comparable pressure.

Fluorescein angiography

Widefield fluorescein angiography (Optos California; Optos plc, Dunfermline, UK) was performed in both eyes. In the right eye, dye first entered the central retinal artery 20.1 seconds after the start of injection and complete filling of the retinal arteries was reached at 25 seconds. Both times were prolonged, and 4.9 seconds elapsed between the first appearance of dye and complete arterial filling. The onset of choroidal filling could not be clearly identified on the images obtained in this case, and choroidal circulation time was therefore not assessed. Numerous microaneurysms were seen from the posterior pole to the mid-periphery, accompanied by widespread vascular leakage in the same area. The retinal capillary network was poorly delineated overall, indicating reduced perfusion; however, no area could be delineated as a discrete zone of capillary nonperfusion. The foveal avascular zone was not appreciably enlarged. No retinal neovascularization was seen (Figure 3).

**Figure 3:**
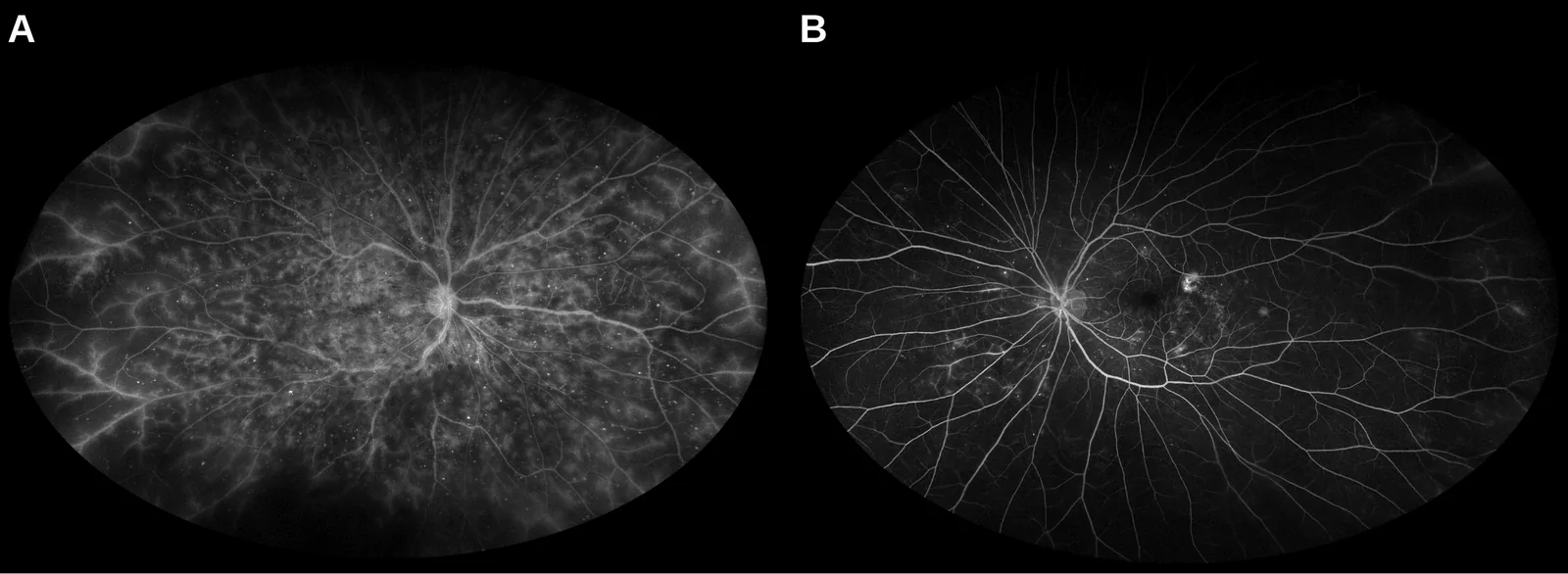
Widefield fluorescein angiography at presentation, mid-phase. (A) The right eye shows numerous microaneurysms from the posterior pole to the mid-periphery, with widespread vascular leakage and reduced perfusion of the retinal capillary network. (B) The left eye shows only a few microaneurysms at the posterior pole, without intraretinal microvascular abnormalities, reduced perfusion, or widespread vascular leakage.

The left eye showed approximately six microaneurysms at the posterior pole and scattered hard exudates from the nasal to the inferior aspect of the optic disc, without retinal neovascularization, intraretinal microvascular abnormalities, reduced perfusion, or widespread vascular leakage; the findings therefore corresponded to mild nonproliferative diabetic retinopathy. Early-phase images were obtained in the right eye only, so the onset of choroidal filling was not assessed in the left eye.

Treatment and course

On day 0, an aflibercept biosimilar 2 mg/0.05 mL (Fuji Pharma Co., Ltd., Toyama, Japan) was injected into the vitreous of the right eye.

Because of the widespread reduction in retinal capillary perfusion with vascular leakage, and because no further endovascular treatment was feasible so that control of ocular perfusion through treatment of the underlying lesion was considered unattainable, panretinal photocoagulation (PRP) was undertaken for the ischemic retina. PRP was delivered with a single-spot multicolor laser (yellow wavelength, 200-µm spot size, 250-ms duration, 150-220 mW) in four sessions on days 13, 25, 46, and 67, for a total of 1,580 burns.

Optical coherence tomography (SPECTRALIS; Heidelberg Engineering, Heidelberg, Germany) was performed on days 0, 25, and 67. On day 25, the cystoid spaces had decreased, and the foveal contour had been restored, indicating that the macular edema had improved although it had not completely resolved. By day 67, the edema had recurred. The day-67 scan was obtained before the fourth session of photocoagulation on the same day (Figure 4).

**Figure 4:**
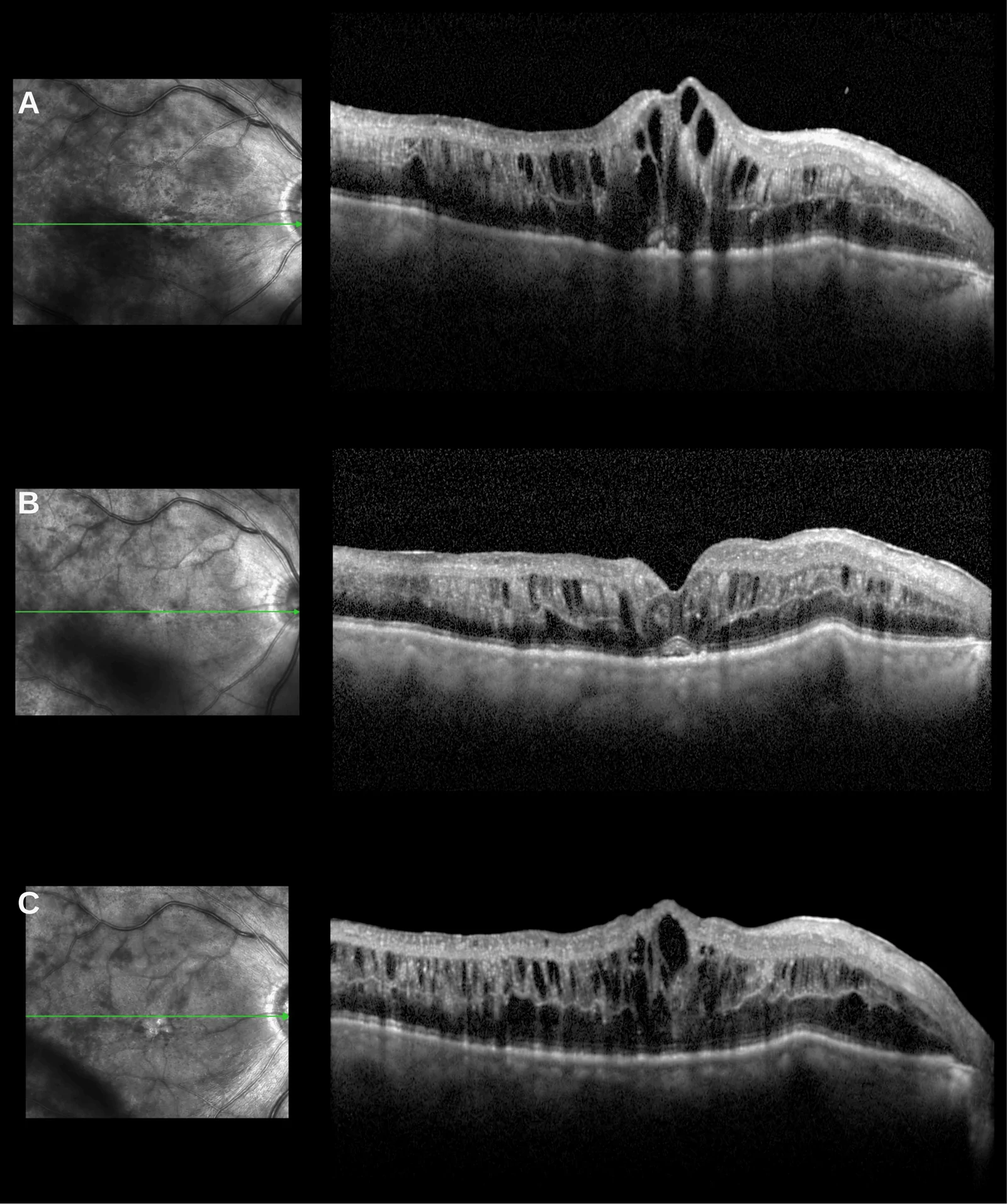
Serial optical coherence tomography of the right eye. Each row shows the infrared image with the horizontal scan line (left) and the corresponding B-scan (right). (A) Day 0, before the first intravitreal injection, showing cystoid macular edema. (B) Day 25, showing a marked reduction of the cystoid spaces and restoration of the foveal contour, with incomplete resolution. (C) Day 67, obtained before the fourth photocoagulation session, showing recurrent macular edema.

BCVA was 0.1 (partial) on day 25 and 0.15 on day 67. Throughout the course at our department, the IOP remained lower in the affected eye than in the fellow eye (affected eye/fellow eye: 14/15 mmHg on day 0, 9.0/13.3 mmHg on day 25, 11.7/15.0 mmHg on day 46, and 11.3/14.7 mmHg on day 67). Undilated slit-lamp examination and gonioscopy showed no iris or angle neovascularization at any visit.

Because the macular edema had recurred, retreatment was planned, and a second injection of the aflibercept biosimilar 2 mg was given on day 77. Retreatment was based on the reappearance of cystoid macular edema. At that visit, undilated slit-lamp examination and gonioscopy again showed no iris or angle neovascularization, and spontaneous pulsation of the central retinal artery persisted as at presentation. Visual acuity, IOP, and optical coherence tomography were not obtained on that day, so the quantitative ocular findings reported here extend to day 67.

## Discussion

After it had been decided that no further endovascular treatment of the causative lesion was feasible, BCVA in the right eye fell from 0.7 (logMAR 0.15) to 0.2 (logMAR 0.70) over approximately two months during which no ocular treatment was given. The two recorded examinations by the referring ophthalmologist document the natural course during an untreated interval.

Previous reports of CRVO associated with CCF all describe the course after the shunt had been closed. These include improvement of the retinal circulation and visual acuity after embolization of external carotid feeders [2], CRVO developing four months after transarterial embolization in a patient whose symptoms had initially improved [3], impending CRVO six months after embolization [4], and CRVO with macular edema after successful embolization that resolved with aflibercept [5]. Improvement in visual acuity and central retinal thickness has also been reported when embolization was combined with an anti-VEGF agent [6]. What distinguishes the present case is that the retinal complication progressed while the shunt persisted and after the option of further endovascular treatment had been lost. To our knowledge, and within the scope of our search, no previous report has described the ophthalmic course of an eye that required parent artery occlusion of the internal carotid artery and in which the shunt nonetheless persisted. When endovascular treatment has been incomplete, ophthalmic follow-up should be continued for as long as the shunt persists. Retinal complications can occur even when angiography shows a reduced shunt volume [4], so angiographic improvement does not mean that ocular complications will not develop.

The ocular findings in this case comprised two sets of signs. Dilated retinal veins, retinal hemorrhages extending from the posterior pole to the periphery, and marked macular edema are findings of CRVO. In contrast, the lack of prominent venous tortuosity, the attenuated retinal arteries, the widespread reduction in retinal capillary perfusion, the numerous microaneurysms from the posterior pole to the mid-periphery, the consistently lower IOP in the affected eye, the prolonged arterial filling on angiography (first appearance of dye at 20.1 seconds and complete filling at 25 seconds), and the spontaneous pulsation of the central retinal artery seen only in the affected eye all suggest reduced ocular perfusion pressure [7].

Spontaneous pulsation of the central retinal artery is particularly informative. It was present only in the affected eye, and no pulsation could be induced in the fellow eye by comparable pressure. Spontaneous pulsation indicates that the diastolic pressure in the retinal artery has fallen close to the IOP; ordinarily, pulsation is seen only when it is induced by digital pressure. Because this was an interocular difference, it cannot readily be attributed to systemic factors such as circulation time or injection conditions. The spontaneous pulsation was still present at the day 77 examination, suggesting that the reduction in ocular perfusion pressure was not a transient phenomenon.

The early phase of the angiogram points in the same direction. Dye first entered the central retinal artery 20.1 seconds after the start of injection and complete arterial filling was reached at 25 seconds, both of which are prolonged; the 4.9 seconds required between these two points also indicates slow arterial inflow. However, early-phase images were obtained only in the affected eye, and these values may be influenced by systemic factors such as circulation time and injection conditions, so they must be interpreted with caution. Prolonged circulation time in the affected eye has also been reported in CRVO itself [8], so a prolonged circulation time alone cannot establish arterial compromise.

The affected eye also showed a reduced pupillary light reflex and poor mydriasis. Because there were no posterior synechiae or iris atrophy and the response to the same mydriatic agent was poor only in the affected eye, a mechanical cause does not account for these findings. The iris is supplied by the long posterior ciliary arteries and the anterior ciliary arteries, both of which arise from the ophthalmic artery, so chronic hypoperfusion of the iris may have contributed. However, damage to the parasympathetic fibers of the oculomotor nerve or to the sympathetic plexus around the internal carotid artery in association with the cavernous sinus lesion cannot be excluded, and the mechanism could not be determined. Diabetic autonomic neuropathy is generally a systemic process and does not readily explain poor mydriasis confined to one eye.

The patient had undergone parent artery occlusion of the right internal carotid artery as part of the treatment of the CCF. The ophthalmic artery arises from the supraclinoid segment of the internal carotid artery, so depending on the extent of the occlusion, ocular perfusion may depend on collaterals from the external carotid system. In that situation, flow travels retrogradely within the ophthalmic artery and the perfusion pressure falls. The superficial temporal artery-middle cerebral artery bypass performed before the parent artery occlusion in this case supplies the middle cerebral artery territory and would not be expected to contribute to the ophthalmic artery. The constellation of findings observed here suggests that ocular perfusion may not have been adequately compensated.

At the same time, the persistence of shunt flow into the right superior ophthalmic vein means that elevated orbital venous pressure may have coexisted as a separate mechanism, and the persistent dilatation of the episcleral vessels supports the clinical operation of that mechanism. However, the IOP in this case remained lower in the affected eye, the opposite of the raised IOP generally reported in CCF as a result of elevated episcleral venous pressure [1]. It is possible that reduced aqueous production from ciliary body hypoperfusion outweighed the rise in venous pressure.

A case has been reported in which cerebral revascularization was performed for ocular ischemic syndrome due to internal carotid artery occlusion, but visual acuity did not improve, showing that reconstruction of the cerebral circulation does not necessarily compensate for the ocular circulation [9]. The authors of that report noted that in patients with internal carotid artery stenosis or occlusion, attention should be paid to ocular as well as cerebral ischemia. In the present case, we did not assess ophthalmic artery blood flow, and because findings of CRVO clearly coexisted, we could not evaluate the arterial contribution independently. We therefore did not arrive at a diagnosis of ocular ischemic syndrome and have described the findings as suggesting reduced ocular perfusion pressure. It should also be noted that an anti-VEGF agent was injected on the day of presentation and PRP was started 13 days later, so the absence of neovascularization during the course may reflect the treatment given and cannot be used to argue for or against that diagnosis. All of the above describe the internal consistency of the findings and do not establish the mechanism. We did not perform orbital ultrasonography to assess flow in the ophthalmic artery or the superior ophthalmic vein, and neither reduced ocular perfusion nor elevated orbital venous pressure was directly confirmed.

The patient was a 63-year-old man with type 2 diabetes mellitus, hypertension (150/90 mmHg), and a history of smoking 20 cigarettes per day from the age of 20-45 years. The diabetes had been untreated for approximately 10 years, and treatment had been restarted after admission for the current injury. All of these are established systemic risk factors for CRVO, and it cannot be excluded that CRVO would have developed in this patient irrespective of the CCF.

Numerous microaneurysms were present from the posterior pole to the mid-periphery in the affected eye. Against a background of long-standing untreated diabetes, microaneurysms are consistent with diabetic retinopathy. The fellow eye also showed approximately six microaneurysms and scattered hard exudates at the posterior pole, indicating that diabetic retinopathy was present in both eyes. However, the fellow eye showed no intraretinal microvascular abnormalities and no reduction in perfusion, and corresponded to mild nonproliferative diabetic retinopathy. In contrast, the affected eye showed no hard exudates and was dominated by numerous microaneurysms with widespread vascular leakage and reduced perfusion, so that the two eyes differed in the nature as well as the severity of the findings. Diabetic retinopathy is a bilateral disease that progresses in a broadly symmetric fashion, and a difference of this degree under the same glycemic control (glycated hemoglobin 7.2% at presentation) is difficult to explain by diabetes alone. The same applies to hypertension and to the smoking history.

Several findings suggest a contribution from local factors. First, the CCF, the parent artery occlusion, and the retinal disease were all confined to the right side. Second, angiography confirmed shunt flow from the residual shunt into the superior ophthalmic vein, providing a mechanism for elevated orbital venous pressure. Third, proptosis and dilated episcleral vessels, indicating orbital congestion, had been present before the findings of CRVO became manifest. Fourth, the spontaneous pulsation of the central retinal artery and the consistently lower IOP in the affected eye both suggested reduced ocular perfusion after parent artery occlusion.

Nevertheless, all of these observations only suggest a contribution from local factors and do not establish causation. The microaneurysms in the affected eye could arise from diabetic retinopathy, from chronic ocular hypoperfusion [7], or from CRVO, and cannot be attributed to a single disease. This report does not claim that the residual shunt or the parent artery occlusion caused the CRVO.

The lesson from this case is that even in a patient with systemic risk factors, a contribution from local factors should be considered when the severity of the findings differs markedly between the two eyes. Assessment of the fellow eye provides a useful internal control for that judgment.

Macular edema was treated with an aflibercept biosimilar. Optical coherence tomography was performed immediately before the first injection and on days 25 and 67. On day 25, the cystoid spaces had clearly decreased, and the foveal contour had been restored, so that the edema had improved although it had not completely resolved; by day 67, it had recurred.

Two confounders complicate the interpretation of this course. First, PRP was delivered throughout the interval after the first injection in four sessions, and PRP itself can worsen macular edema. The day-67 scan was obtained before the fourth session on the same day, but it was taken 21 days after the third session, so the contribution of photocoagulation cannot be separated from the natural course. Second, neither the hemodynamics of the residual shunt nor ocular perfusion after parent artery occlusion was assessed.

For PRP, no randomized trial exists for eyes with CCF, so the evidence must be extrapolated from studies of retinal vein occlusion. The Central Vein Occlusion Study Group N report found that prophylactic PRP for ischemic CRVO did not completely prevent iris or angle neovascularization and recommended frequent follow-up with prompt photocoagulation when neovascularization develops. It also reported that regression within one month after photocoagulation for neovascularization occurred in 18 of 32 untreated eyes (56%) but in only four of 18 eyes that had received prophylactic PRP (22%) [10]. In the present case, photocoagulation was performed in view of the widespread reduction in retinal capillary perfusion with vascular leakage and of the fact that further treatment of the causative lesion was not feasible. We did not classify the perfusion status of this eye, and photocoagulation was therefore not undertaken after confirming that the conventional criteria for the ischemic type had been met.

No iris or angle neovascularization was observed during the reported period. This does not mean that the anti-VEGF agent or the PRP prevented neovascularization, because both were given early in the course and the observation period was only 67 days. In CRVO, 34% of eyes judged to be perfused convert to nonperfusion within three years, and nonperfusion has been reported to progress continuously throughout follow-up [11]. Undilated examination of the iris and the angle should therefore be continued.

Most importantly, ophthalmic treatment corrects neither the hemodynamics of the residual shunt nor ocular perfusion after parent artery occlusion. The anti-VEGF agent and the PRP used in this case were symptomatic interventions directed at the secondary macular edema and the risk of neovascularization, and the disease must be assessed with an understanding of both the effects and the limitations of these treatments. Systemic management of the diabetes and hypertension is also important, including for prevention of disease in the fellow eye.

Limitations

This report has several limitations. It describes a single case, and only one injection of the aflibercept biosimilar was given during the reported period, so the treatment response was assessed from a single administration; in addition, panretinal photocoagulation was delivered throughout the same interval, and its contribution to the observed recurrence cannot be separated from the natural course. The completeness of photocoagulation coverage was not confirmed with repeat angiography. The extent of the parent artery occlusion and the source of blood supply to the ophthalmic artery after occlusion were not assessed, so the vessels supplying ocular perfusion cannot be determined, and orbital ultrasonography of the ophthalmic artery and the superior ophthalmic vein was not performed, so neither reduced ocular perfusion nor elevated orbital venous pressure was directly confirmed. Early-phase angiographic images were obtained only in the affected eye, so the timing of dye entry into the retinal arteries was not compared with the fellow eye, and the onset of choroidal filling could not be clearly identified, so choroidal circulation time was not assessed. Fluorescein angiography was performed with widefield imaging, and the field did not adequately include the retina anterior to the equator, so the area of capillary nonperfusion was not quantified and direct comparison with the conventional criteria for the ischemic type, which are based on standard seven-field angiography, is not possible [12]; the findings are therefore described as reduced retinal capillary perfusion with vascular leakage rather than as discrete areas of nonperfusion. The angiographic findings of the fistula are based on the report from the referring institution and were not independently reviewed by the authors, and the state of the shunt during ophthalmic follow-up was not assessed. The patient had several systemic risk factors for central retinal vein occlusion, so a contribution from local factors cannot be established and coincidental coexistence cannot be excluded. Finally, the observation period was short and did not extend beyond the period of highest risk for iris and angle neovascularization; visual acuity, intraocular pressure, and optical coherence tomography were not obtained on day 77 when the second injection was given, so the quantitative ocular findings extend only to day 67.

## Conclusions

We have reported a case in which the shunt persisted after endovascular treatment of a traumatic CCF and parent artery occlusion of the internal carotid artery was required, and in which CRVO accompanied by findings suggesting reduced ocular perfusion developed with rapid visual loss. When endovascular treatment has been incomplete, ophthalmic follow-up and treatment should be continued for as long as the shunt persists; in this case, visual acuity deteriorated without ocular treatment during the approximately two months after it had been decided that no further endovascular treatment was feasible.

Even in a patient with systemic risk factors, a contribution from local factors should be considered when the severity of the findings differs markedly between the two eyes, and assessment of the fellow eye provides a useful internal control. In an eye that has undergone parent artery occlusion, reduced ocular perfusion pressure should be assessed in addition to the venous pathology. Interocular comparison of spontaneous pulsation of the central retinal artery, the interocular difference in intraocular pressure and its course, the degree of retinal venous tortuosity, attenuation of the retinal arteries, the timing of dye entry into the retinal arteries on early-phase angiography, and angiography of the fellow eye allowed the arterial findings in this case to be documented without orbital ultrasonography or ophthalmic artery angiography. Ophthalmic treatment corrects neither the hemodynamics of the residual shunt nor ocular perfusion after parent artery occlusion. It is a symptomatic intervention, and the disease must be assessed with an understanding of both the effects and the limitations of treatment.
